# *Saline Systems *highlights for 2006

**DOI:** 10.1186/1746-1448-3-1

**Published:** 2007-01-23

**Authors:** Shiladitya DasSarma

**Affiliations:** 1 University of Maryland Biotechnology Institute, Center of Marine Biotechnology, Baltimore, Maryland 21202, USA

## Abstract

*Saline Systems *is a journal devoted to both basic and applied studies of saline and hypersaline environments and their biodiversity. Here, I review the reports and commentaries published in the journal in 2006, including some exploring the geochemistry of saline estuaries, lakes, and ponds, others on the ecology and molecular biology of the indigenous halophilic organisms, and still others addressing the environmental challenges facing saline environments. Several studies are relevant to applications in biotechnology and aquaculture.

## Introduction

Saline environments and biodiversity associated with such environments are challenged from both human activities and natural dynamics. For example, recent climatic changes have led to increasingly intense monsoon rains in some regions and to decreasing precipitation levels in others. Nowhere are such changes more in evidence than in the salinity characteristics of many coastal estuarine bodies and inland saline lakes. *Saline Systems *specializes in meeting the publication needs of researchers working in such saline environments and provides an interdisciplinary and readily accessible forum for scientists worldwide [1]. In 2006, *Saline Systems *has published a wide variety of important studies on saline environments, including detailed analyses of halophilic species inhabiting them, and those important to biotechnology and aquaculture.

## Geochemical studies of saline environments

Four reports in *Saline Systems*, volume 2 established baseline hydrogeochemistry for several geographically dispersed saline ecosystems. The paper by Weber et al. [2] provided perspective on the effects of more than a century of human activity on Fleet, Dorset, a coastal lagoon in the southeast of Britain, using modeling of historical data on land use, livestock, and human population. The study found that livestock were the main nutrient source throughout, but inputs from inorganic fertilizers were substantial in the modern period, and an ancient swan community also contributed to the on-going environmental challenges. The authors concluded that a combination of measures is essential to address these challenges.

Two studies reported on salinity and hydrological studies of salt ponds in islands, the British Virgin Islands in the Caribbean [3] and Christmas and Washington Islands in the Pacific [4]. The study of Jarecki and Walkey [3] found that salt ponds were increasingly threatened by rapid coastal fluctuations and isolated from the sea, exhibiting dramatic fluctuations in salinity in response to rainfall and evaporation. The study of Saenger et al. [4] examined a series of lakes which present a modern analog to the chemical and morphological evolution of isolated basins. Their findings may be correlated to shifts in climate such as El Niño. The patterns of variation and succession in these studies are applicable to salt pond management globally.

An interesting study by Alipour [5] reported on the chemical composition of the large hypersaline Urmia Lake in Iran, which has been designated as an international park by the United Nations. The study found that the lake has been experiencing significant changes over the past decade as a result of climatic and human effects (e.g. reduced precipitation and on-going construction of a causeway). This study offers one of the first opportunities for a detailed chemical picture of this important lake and its comparison to other large hypersaline environments.

## Biology of organisms in saline environments

Five reports in *Saline Systems*, volume 2 provide insights into the biology of microorganisms in saline environments and ways in which they cope with environmental challenges. One report [6] examined the natural communities of square archaea in artificial saltern crystallizer ponds in Eilat, Israel. While most other halophilic archaea produce intracellular buoyant gas-filled vesicles for floatation, the studies of Oren et al. suggested that cells in natural communities contain too few vesicles to serve this function. They propose that the presence of vesicles close to the cell periphery may orient cells perpendicular to incident sunlight and increase the efficiency of light harvesting by bacteriorhodopsin in their membrane.

Another report, by Kurz et al. [7], addressed the role of the NhaD family of Na^+^/H^+^-antiporters in the salt response of the extremely halophilic bacteria, *Halomonas elongata*. The characterization of the gene product by complementation of salt sensitive *E. coli *and the distribution of this gene in bacteria indicated that NhaD may function in Na^+^-import for osmoregulation in marine habitats.

In a study on the effect of cyanobacteria on salinity tolerance and productivity of rice, Rodríguez et al. [8] examined the role of extracellular products of the cyanobacterium *Scytonema hofmanni *in comparison to gibberellic acid. Evaluation of growth and biochemical parameters showed that the negative effect of salt exposure (with the exception of chlorophyll, which increased) was nullified by extracellular products. Exposure to either extracellular products or gibberellic acid resulted in a reversal of some of the responses to salt stress. The authors proposed that *S. hofmanni *extracellular products may counteract altered hormone homeostasis of rice seedlings under salt stress by producing gibberellin-like plant growth regulators.

A study by Crowley and co-workers [9] addressed the genetic requirements for UV tolerance in halophilic archaea. This work was a follow-up study to one previously published in *Saline Systems *which reported a whole-genome microarray analysis of the response of *Halobacterium *sp. NRC-1 to UV irradiation [10]. In the latter work, the bacterial-type *uvrA*, *uvrB*, and *uvrC *nucleotide excision repair genes were deleted and shown to result in increased UV sensitivity, which correlated to the lack of removal of cyclobutane pyrimidine dimers or 6–4 photoproducts in the dark. However, the new study also found that the increased sensitivity of the *uvr *mutants is greatly attenuated in visible light, confirming the importance of photoreactivation in this organism. They also report the complex ancestry of the *uvr *genes in archaea.

A general review of post-genomic studies on *Halobacterium *sp. NRC-1, which has become a model organism for understanding life in hypersaline environments, was presented by DasSarma et al. [11]. Since its genome sequence was completed in 2000, a combination of genetic, transcriptomic, proteomic, and bioinformatic approaches have provided insights into both its extremophilic lifestyle and fundamental cellular processes. The article reviews post-genomic research on DNA replication and repair systems, phototrophic, anaerobic, and other physiological capabilities, the proteome of the organism, which is extremely acidic, and role of lateral gene transfer in its evolution.

## Environmental challenges facing saline environments

Four articles in *Saline Systems*, volume 2 documented some of the problems confronting biodiversity in saline environments. An article by Carmichael and Li [12] reported on the correlation between cyanobacterial toxins and large mortalities of the eared grebe observed at the Salton Sea in recent years. This is an important environment for migratory birds, and the largest inland body of water in California, which was formed via diversion of the Colorado River in the early 20^th ^century. The reported results of phytoplankton identification and cyanotoxin analysis found that microcystins (which are potent liver toxins) result from indigenous cyanobacteria, e.g. *Synechococcus *and *Oscillatoria *species. The levels of microcystins found in affected grebe livers were high enough to explain some of the acute mortalities. The authors recommend that future efforts should evaluate ways to control and mitigate toxic cyanobacterial waterblooms at the Salton Sea.

Velasco et al. [13] reported on a study of primary producers and macroinvertebrate communities in a Mediterranean hypersaline stream in Spain which has experienced large dilutions from irrigation. The study included species surveys along the salinity gradient, in both runs and pools. Although diversity indices decreased with increasing salinity, the abundance and evenness indices did not show significant changes to salinity. The authors concluded that salinity was the most important factor for community composition and structure in the stream.

Another study, reported by Castro et al. [14], focused on populations of the brine shrimp *Artemia *from a variety of diverse lakes in Mexico and Chile for evaluation of their aquaculture potential. Their study includes a database of biological data and also ecological data, which will serve as a valuable baseline for future studies of *Artemia *biodiversity. The report also underscored the need to integrate regional information to tackle fundamental problems underlying practical utilization of *Artemia*.

A commentary by Gajardo et al. [15] addressed the importance of *Artemia *as a model of biodiversity analysis at the population level. The authors provided an argument for how a unique opportunity is afforded for understanding biodiversity at the intra-species level in a low diversity ecosystem, with relevance to social and economic benefits. They also emphasized that the *Artemia *system permits experimental testing in both laboratory scale and outdoor pond systems on a global scale.

## Meeting review

A review of the XI International Rotifer Symposium held in Mexico was provided by Sarma [16]. The themes of the conference included rotifer research from morphology to molecular biology, both basic ecological studies and applied areas, including ecotoxicology and aquaculture.

## Epilog

The past year, 2006, was the first complete year for *Saline Systems *and an exciting follow-up to our inaugural year, 2005. In 2007, we invite submissions of high quality manuscripts on all aspects of halophiles and saline environments.
